# Environmental drivers of *Ixodes ricinus* abundance in forest fragments of rural European landscapes

**DOI:** 10.1186/s12898-017-0141-0

**Published:** 2017-09-06

**Authors:** Steffen Ehrmann, Jaan Liira, Stefanie Gärtner, Karin Hansen, Jörg Brunet, Sara A. O. Cousins, Marc Deconchat, Guillaume Decocq, Pieter De Frenne, Pallieter De Smedt, Martin Diekmann, Emilie Gallet-Moron, Annette Kolb, Jonathan Lenoir, Jessica Lindgren, Tobias Naaf, Taavi Paal, Alicia Valdés, Kris Verheyen, Monika Wulf, Michael Scherer-Lorenzen

**Affiliations:** 1 Geobotany, Faculty of Biology, University of Freiburg, Schänzlestr. 1, 79104 Freiburg, Germany; 2 Institute of Ecology and Earth Sciences, University of Tartu, Lai 40, 51005 Tartu, Estonia; 3 Black Forest National Park, Kniebisstraße 67, 77740 Bad Peterstal-Griesbach, Germany; 4 Natural Resources & Environmental Effects, IVL Swedish Environmental Research Institute, Box 210 60, 100 31 Stockholm, Sweden; 5 Southern Swedish Forest Research Centre, Swedish University of Agricultural Sciences, Box 49, 230 53 Alnarp, Sweden; 6 Landscape Ecology, Department of Geography and Quaternary Geology, Stockholm University, 106 91 Stockholm, Sweden; 7 DYNAFOR, Université de Toulouse, INRA, INPT, Chemin de Borde Rouge, CS 52627, 31326 Castanet, France; 8 UR “Ecologie et Dynamique des Systèmes Anthropisés” (EDYSAN, FRE 3498 CNRS-UPJV), Jules Verne University of Picardie, 1 rue des Louvels, 80037 Amiens Cedex 1, France; 9 Forest & Nature Lab, Ghent University, Geraardsbergsesteenweg 267, 9090 Melle-Gontrode, Belgium; 10 Department of Plant Production, Ghent University, Proefhoevestraat 22, 9090 Melle, Belgium; 11 Faculty of Biology/Chemistry (FB 02), Institute of Ecology, Vegetation Ecology and Conservation Biology, University of Bremen, Leobener Str. 5, 28359 Bremen, Germany; 12 Institute of Land Use Systems, Leibniz-ZALF (e.V.), Eberswalder Str. 84, 15374 Müncheberg, Germany; 13 UF PRiMAX, Clinical Pharmacology Department, CHU Amiens-Picardie, 80054 Amiens Cedex 1, France

**Keywords:** Climate gradient, Ecological niche, Ecosystem disservice, Functional ecology, Habitat composition, Landscape composition, Land-use change, smallFOREST, Tick distribution

## Abstract

**Background:**

The castor bean tick (*Ixodes ricinus*) transmits infectious diseases such as Lyme borreliosis, which constitutes an important ecosystem disservice. Despite many local studies, a comprehensive understanding of the key drivers of tick abundance at the continental scale is still lacking. We analyze a large set of environmental factors as potential drivers of *I. ricinus* abundance. Our multi-scale study was carried out in deciduous forest fragments dispersed within two contrasting rural landscapes of eight regions, along a macroclimatic gradient stretching from southern France to central Sweden and Estonia. We surveyed the abundance of *I. ricinus,* plant community composition, forest structure and soil properties and compiled data on landscape structure, macroclimate and habitat properties. We used linear mixed models to analyze patterns and derived the relative importance of the significant drivers.

**Results:**

Many drivers had, on their own, either a moderate or small explanatory value for the abundance of *I. ricinus*, but combined they explained a substantial part of variation. This emphasizes the complex ecology of *I. ricinus* and the relevance of environmental factors for tick abundance. Macroclimate only explained a small fraction of variation, while properties of macro- and microhabitat, which buffer macroclimate, had a considerable impact on tick abundance. The amount of forest and the composition of the surrounding rural landscape were additionally important drivers of tick abundance. Functional (dispersules) and structural (density of tree and shrub layers) properties of the habitat patch played an important role. Various diversity metrics had only a small relative importance. Ontogenetic tick stages showed pronounced differences in their response. The abundance of nymphs and adults is explained by the preceding stage with a positive relationship, indicating a cumulative effect of drivers.

**Conclusions:**

Our findings suggest that the ecosystem disservices of tick-borne diseases, via the abundance of ticks, strongly depends on habitat properties and thus on how humans manage ecosystems from the scale of the microhabitat to the landscape. This study stresses the need to further evaluate the interaction between climate change and ecosystem management on *I. ricinus* abundance.

**Electronic supplementary material:**

The online version of this article (doi:10.1186/s12898-017-0141-0) contains supplementary material, which is available to authorized users.

## Background

The castor bean tick *(Ixodes ricinus)* acts as vector for several infectious diseases (Lyme borreliosis, Tick-borne Encephalitis, Babesiosis etc.) that pose a risk to live stock and human health [1, 2]. Ultimately, the prevalence of tick-borne diseases (TBDs) constitutes an important ecosystem disservice [3, 4], which plays a major role for public health in Europe [5]. Shifts in the distribution of *I. ricinus* and the pathogens it transmits have been observed in the wake of climate and land-use change [6, 7]. In response to these changing conditions a detailed understanding of *I. ricinus* abundance patterns is necessary to predict and reduce exposure risk to TBDs and adjoining monetary expenses for humans.

**Fig. 1:**
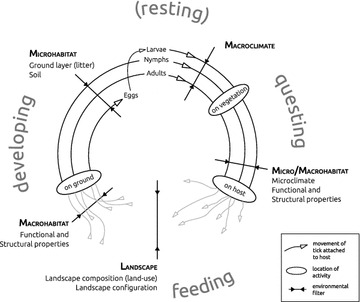
Tick life cycle in a specific habitat patch with particular emphasis on the driver groups analysed in this study. Ticks go through different activity modes (*gray*, *large font*) within their life-cycle, while various environmental filters act upon them. While feeding they are attached to their host and get transported to wherever the host moves. Environmental filters also act upon tick-hosts, indirectly determining the success of ticks. Following this logic, the environment relevant for ticks also comprises the host surface and its properties (e.g. fur density or body size), which may thus be microhabitat drivers during the ‘on host’ phase with a certain influence on the success of ticks. This is however omitted from the graph, because it goes beyond the scope of this study

*Ixodes ricinus* has a life-cycle consisting of three mobile ontogenetic stages (larvae, nymphs and adults), into which it develops immediately after a successful blood meal from vertebrate hosts [8]. Adult ticks are male or female and if they encounter each other on a suitable host and the female tick acquires a last blood meal and falls of the host in a random location, it may deposit eggs from which new larvae can hatch. The ecology of *I. ricinus* is complex with each ontogenetic stage having its own multitude of driving factors (Fig. 1) [9]. Many studies shed light on these factors [10], but they either consider a narrow spatial extent [11, 12] or encompass a small subset of known tick abundance drivers [13, 14]. While these earlier studies help to provide a general overview of the realized niche of *I. ricinus*, there is still a lack of large-scale studies, where the majority of environmental factors potentially driving tick abundance are evaluated simultaneously [9]. Here, we identify a set of relevant drivers of *I. ricinus* abundance to shed light on its realized niche. We surveyed deciduous forest fragments (hereafter ‘habitat patch’) of different sizes and age in rural landscapes, which are very common in Central Europe [15]. The extensive spatial and climatic gradient ranging from southern France to Estonia and central Sweden covers a extensive part of the *I. ricinus* distribution range.

Macroclimate is an important determinant of the *I. ricinus* distribution [16]. Ticks are not able to persist below or above certain climatic thresholds of temperature and relative humidity, because freezing or desiccation threatens their survival [17]. Within the suitable range of these macroclimatic factors, temperature sums (the accumulated temperature over a given period of time) drive tick development rates [17] and the potential abundance of questing ticks [6]. However, the forest canopy buffers macroclimatic conditions considerably [18], which means that effects of macroclimate and tree/shrub abundance are confounded. Because of that and since ticks are relatively immobile horizontally [19, 20], the habitat specific vertical profile of microclimate can be assumed to drive tick abundance [21].

Animals such as ungulates, birds and small mammals are amongst the most relevant tick-hosts [10]. Landscape composition and configuration [22–24] as well as biotic interactions [25, 26] are important determinants of tick-host communities at regional and local scales [27], albeit exact effects on tick abundance remain unclear (Fig. 1). However, cascading effects of landscape factors, as proxy for prevailing tick-host communities, have previously been shown to be relevant for *I. ricinus* abundance [28–30]. Not only landscape properties, but also the properties of the habitat itself determine local visitation rates by tick-hosts (Fig. 1). Morphological plant traits, as well as the structural complexity of a habitat patch determine the availability of shelter for tick-hosts [24, 27] whereas plant functional traits determine the availability of food for many tick-hosts [22]. However, the properties of a habitat patch may be confounded with spatial or temporal continuity of the patch. Ancient forests, for instance, often have higher structural [31] and functional diversity [32, 33]. Similarly, larger patches have a higher chance of including distinct sub-stands resulting in more diverse patch properties. Hence, both older as well as larger patches may provide a larger amount of distinct niches and food resources for tick-hosts. This potentially increases the abundance and modifies the community composition of tick-hosts, presumably with cascading effects on tick abundance [14].

Tick activity is determined by the herb layer as questing habitat, in terms of its cover [11, 12] and vertical structure [12, 34, 35] and by the abundance of the leaf-litter layer as moulting or resting habitat [36] (Fig. 1). Therefore, biotic conditions of the habitat should be considered as limiting drivers, influencing the questing and survival success of *I. ricinus* considerably. Suitable conditions may lead to an increase in feeding opportunities for ticks, thereby raising the likelihood of life-cycle completion and thus tick survival and abundance. The link between the different ontogenetic stages and their abundance has so far not been sufficiently addressed. Younger tick stages are more prone to desiccation than older stages [36]. They utilize different strata of the microhabitat, which is partly an adaption to the different microclimatic requirements of the different ontogenetic stages. This differentiated utilization of the microhabitat results in access to only a stage-specific fraction of the overall host community [34]. Consequently, a distinct influence of environmental factors and tick-hosts on the abundance of the different ontogenetic stages can be expected. Ultimately, these aspects can be interpreted as ontogenetic niche differentiation for *I. ricinus* [37]. However, it is not clear which fraction of a given ontogenetic stage has access to and hence depends on local or regional hosts. Albeit younger stages generally depend more on local hosts and older stages more on regional hosts [8], there may be local habitat properties, as outlined above, which attract the different stages’ hosts differently. This may result in an unexpected local accumulation or depletion of a given ontogenetic stage. In any case, cascading effects of younger on older ontogenetic stages, mediated by common drivers, may exist. By testing the influence of the previous stage, we can test the degree to which drivers of the previous ontogenetic stage (explaining its abundance) influence the stage in focus, implying theoretical cascading effects.

As an overarching objective, we quantify the relative explanatory value of each of the significant drivers of *I. ricinus* abundance, when all the significant drivers are simultaneously retained in one model per ontogenetic stage. With the help of a multi-factorial model we answer these following research questions:

1. Do macroclimatic metrics explain tick abundance when habitat characteristics that buffer macroclimate are accounted for?
2. Does (a) landscape configuration, in terms of fragmentation, lead to higher tick abundances in rural landscapes and does (b) landscape composition, in terms of proportion of more intensive land-use types of the non-forest matrix, indirectly drive *I. ricinus* abundance?
3. Do (a) structural and functional properties of the habitat patch affect tick abundance and do (b) more diverse conditions in these properties or higher temporal continuity of the patch lead to higher tick abundances?
4. Are (a) the different ontogenetic stages driven to a varying degree by different drivers and (b) how important are common drivers, in terms of abundance of the previous ontogenetic stage for *I. ricinus*?

## Methods

### Study locations

**Fig. 2:**
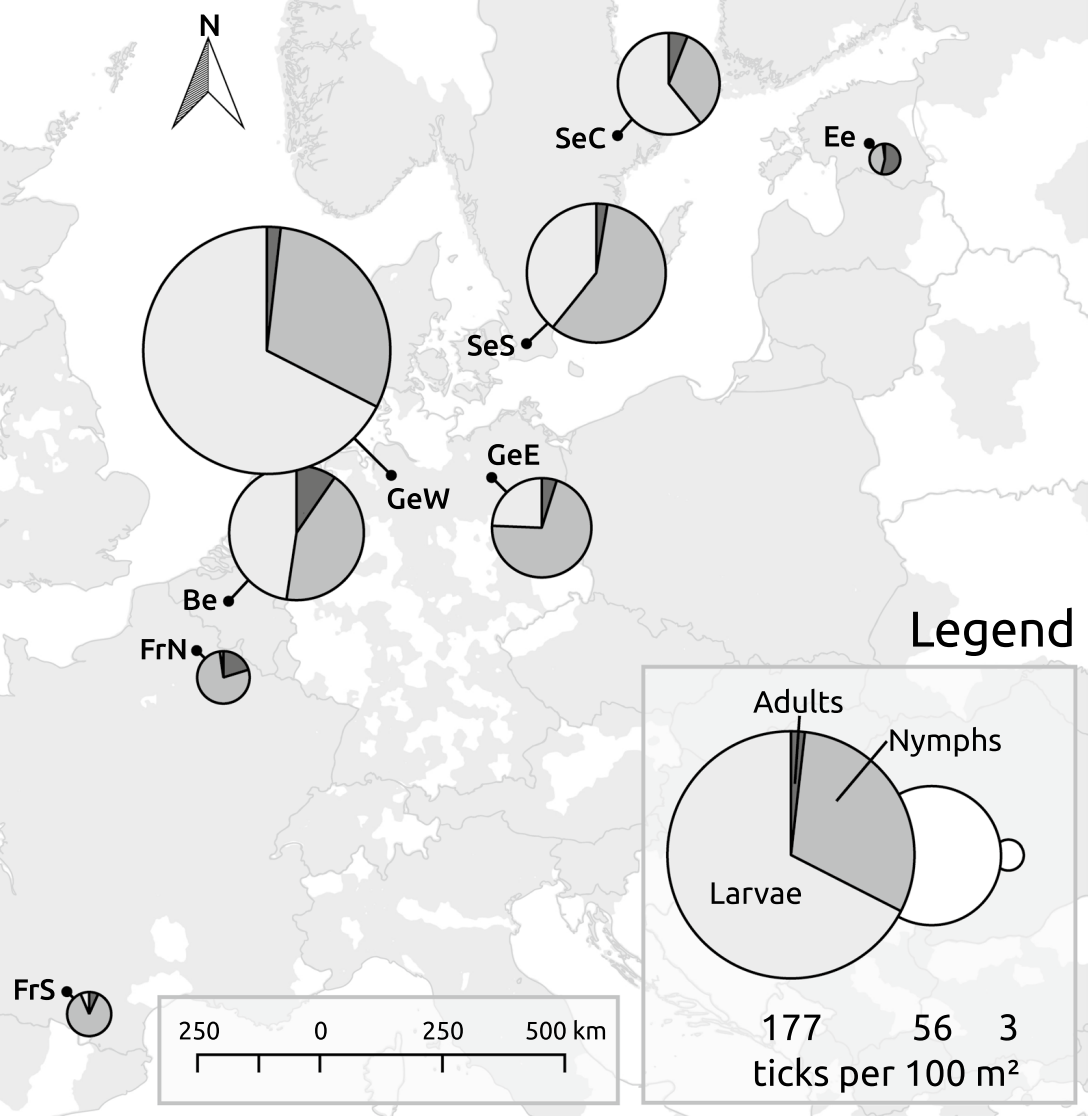
Distribution of collected ontogenetic stages in all eight sampled regions. Size of the circle corresponds to the average abundance of ticks collected per 100 m^2^. All plots sampled for tick abundance were included to derive average values, also those plots where we did not find any ticks. The *gray area* on the map indicates the known distribution of *I. ricinus* in January 2016 (taken from [72])

This study was carried out within the framework of the smallFOREST project [38]. Study sites were located in eight regions across the temperate zone of Europe (southern and northern France, Belgium, western and eastern Germany, southern and central Sweden and Estonia, Fig. 2). Two landscape windows each of 5 km × 5 km extent, but with contrasting landscape configuration and composition due to differences in agricultural disturbance intensities, were selected in each of the eight regions. These two landscape windows were selected to be representative of the regional gradient in land-use intensity. In each of these 16 windows a total of 14–16 forest patches (depending on local availability) of different size and age were selected as focal forest patches. Forest patches had to be dominated by deciduous tree species covering more than 60% of the tree layer to be considered for sampling. Our selection resulted in a total of 250 forest patches (Additional file 1). Sampling was confined to a random subset of predetermined plots, as described in [38]. The plots were distributed regularly within each patch, their number depending largely on patch size (one to 128 plots per patch, on average 5.0). The study design is illustrated in more detail in Fig. 1 of [38].

Sampling of tick and forest stand characteristics was carried out by the same team in all regions whereas soil and vegetation surveys were done by site-specific expert groups.

### Ecological surveys

The major setup of our surveys is designed to capture the key drivers as suggested by [9]. We modified it to consider specific properties of rural landscapes and forest as habitat type. We looked at the driver groups Macroclimate, Landscape and Habitat quality and distinguished, in contrast to [9], between macrohabitat (overstory) and microhabitat (understory vegetation, leaf-litter layer and soil) and considered a potential link between ontogenetic stages (Additional file 2). Specific indicator traits within each driver group were selected to describe different aspects and to be ecologically meaningful for as many as possible functional guilds related to ticks (such as ticks themselves, potential hosts, plants and leaf litter as habitat).

#### Tick survey

A random subset of the predefined plots depending on patch size (between one and nine plots per patch, on average 2.3), was sampled for ticks. We collected ticks at all mobile stages (larvae, nymphs and adults) by drag sampling [39] in 2013 within 1 week per landscape window (Additional file 1). Due to the high number of plots and their spatial distribution, sampling was possible only once in each plot. Drag sampling was done using a 1 m × 1 m piece of white flannel cloth, attached to a wooden stick as handle and a metal chain was attached at its bottom to increase the contact probability between the cloth and the ground-layer. Sampling was carried out only during rain-free day time between 09:00 and 21:00 h.

The cloth was dragged upright through the ground-layer vegetation along four different 25 m long transects within each plot (resulting in a 100 m^2^ sample area). Attached ticks were picked off the cloth after every transect and preserved in ethanol. They were counted later in the laboratory and were determined morphologically to species level according to [40]. Small numbers of *Dermacentor* sp. were encountered, particularly in southern France, but only *I. ricinus* received further consideration. Tick counts per 100 m^2^ were averaged over all plots within one forest patch. Subsequently the averages were log-transformed, using the formula ${\text{x}} ^{\prime} = { \log }_{ 10} ({\text{x}} + 1).$

#### Vegetation survey

The same subset of plots as for the tick sampling was surveyed for forest stand structure (Additional file 1). For each plot we recorded the tree species, height and number of stems, diameter at breast height (D_130_), the distance and azimuth direction for each tree from the plot center and whether the tree was dead or alive. Distance and height measurements were performed using a Vertex IV Hypsometer (Haglöf Inc.). Sampling was restricted to a 20 m radius from the plot center.

Additionally, plant species composition was surveyed during the 2012 and 2013 growing seasons in the same plots with emphasis on the presence and abundance of all plant species. These estimations were performed separately for the herb, shrub and tree layers, by assigning one of the abundance categories 1 (<5 individuals), 2 (5 individuals—30%) or 3 (>30%) to each of the recorded plant species [38].

We derived structural and functional properties at the plot level from the forest stand and vegetation data. We derived stand height, tree density, basal area, tree slenderness coefficients and diameter distributions to describe the structural properties of each forest stand [41]. To capture structural diversity, we calculated the coefficient of variation (for log-normal data) of the tree diameters and of the potential height of the herb layer, the latter derived from the TRY database [42]. Using both, the TRY- [42] and LEDA [43] databases on the vegetation abundance data, we derived functional traits. Based on the detected tree species we derived metrics that are related to the leaf economics spectrum (i.e. traits determining amongst others the decomposition of leaf-litter) [44] (Additional file 2). For herb species growth and life forms, branching types and specific leaf area were determined (all defined in [43]), because they were assumed to influence the suitability of the herb layer as questing habitat for ticks. We determined the richness of different weight-classes of dispersules (lightweight: <0.1 g, medium: 0.1–2 g, heavy: >2 g) [43, 45] and the average overall dispersule mass, separately for all vegetation layers. This served as a proxy for the quality and amount of high energy food potentially available for different tick-hosts, feeding on these dispersules [46]. We weighted tree leaf traits by the summed diameter per tree species and all other traits were weighted by the specie’s abundance to calculate community-weighted means (CWM) of these traits. These CWM values were then averaged over all plots per patch. Plant species diversity was estimated for the herb-, shrub- and tree layers as average species richness (i.e. α-diversity) over all plots per patch. To describe the overall diversity we calculated for each vegetation layer γ-diversity per patch and β-diversity (1-(plot-scale diversity/patch-scale diversity)) as between scale variability [38] (Additional file 2).

#### Soil survey

Soil samples were collected between July and October 2012 before leaf fall so that mostly leaves of the previous growth period were part of the leaf litter layer. The subset of plots selected for this survey differed from the tick/stand structure survey and were independent from the latter. Soil was sampled in between three and 31 plots per patch (on average 6.0). In each plot an area of 25 cm × 25 cm of the forest floor was sampled according to the method described by [47]. After collecting the forest floor material, the topmost 10 cm of mineral soil was sampled using a soil corer with a diameter of 4.2 cm. The forest soil layers were analyzed in the laboratory to determine carbon, nitrogen, phosphor (organic, inorganic and total), ratios thereof and pH (Additional file 3).

#### Landscape metrics

We extracted landscape metrics at the patch and landscape scale (Additional file 3). At the patch scale we determined the size and age of all forest patches and the area covered by edge habitat (using buffers of 5, 10 and 20 m into the patch). At the landscape scale, we determined landscape composition in the form of proportion of different land-use types (forest, arable land, pasture) in concentric buffers (Additional file 3). Fragmentation is quantified in the form of length per hectare (density) of hedgerow and patch edge, the proximity index and distance of the nearest neighbor forest patch (NND). Additionally we determined the amount of edge habitat inside forest patches (as above) in concentric buffers around focal patches (Additional file 3).

#### Climate

We recorded ambient microclimate at the same time as the tick/stand structure survey with Testo 175-H2 Data-Loggers (temperature precision = ±0.5 °C, relative humidity accuracy = ±3%). Measurements were taken every minute for approximately half an hour in the plot center. Air temperature and relative humidity were measured at both 5 and 130 cm height. Soil temperature was measured at a depth of 5 cm. We calculated saturation deficit according to [36], based on values averaged between 5 and 130 cm height for both, relative humidity and air temperature.

Macroclimate data were extracted from the “Global Summary of the Day” (GSOD) dataset hosted on the web-servers of NOAA’s National Centers for Environmental Information (NCEI). We extracted climate metrics for the period from 1st of January 2013 to the day of sampling and for 30 days prior to tick sampling. Additionally, we calculated growing (above 8 °C) and chilling (below 8 °C) degree days from the beginning of 2013 to the day of sampling. All metrics were averaged over all climate stations within a 20 km radius of the landscape window (mostly two but sometimes only one station was available).

### Statistical analyses

All statistical work was carried out in R version 3.3.1 [48]. An explanatory factor analysis (EFA) was carried out based on maximum likelihood to derive factors of correlated variables (Additional files 4, 5) within variable groups with the ‘psych’ package [49]. These correlation factors are assumed to represent the combined (and more general) influence of a set of correlating variables, which would not be significant separately because they might be too specific.

We built three linear mixed models (LMMs) to explore the effect of all environmental variables on the abundance of the three ontogenetic stages (larvae, nymphs and adults) using the ‘lme4’ package [50]. We included the preceding stage’s abundance to consider and compare indirect effects of the drivers of the previous stage relative to the other environmental drivers. Both ‘region’ and ‘window’ were implemented as random factor (two windows nested in each of eight regions =16 levels) to account for the nested structure of our sampling design. As several of the tested variables (such as latitude and longitude or nearest neighbor distance and the proximity index) could explain spatial patterns which would be detected as spatial autocorrelation, given they would be meaningful, while accounting for other significant variables, we did not test for spatial autocorrelation in advance of model-building.

Our standardized, semi-automatic model-building/variable selection procedure is described in Additional files 6 and 7. To capture optimum (i.e. hump-shaped) or pessimum (i.e. u-shaped) conditions in drivers, we also tested second-order polynomials of the variables. The so-derived models were eventually fit using restricted maximum likelihood (REML). The ‘lmerTest’ package [51] was used to determine type-III ANOVA tables with ‘Wald F-Test with Satterthwaite degrees of freedom’ for fixed effects. Response profiles based on partial residuals were determined with the ‘visreg’ package [52] and plotted with the ‘ggplot2’ package [53]. We derived the relative importance as proportion of each significant driver of the overall variation, based on partial eta^2^ values (Additional file 8, [54]). Due to the large number of known effects on tick abundance, it can be expected that several of the significant drivers are small or moderate in their individual importance. Detailed R-code can be found in [55].

## Results

### Tick survey

We collected a total of 24,479 ticks in all stages, of which 12,396 (50.6%) were larvae, 10,992 (44.9%) nymphs and 499 (4.5%) adults. Among adults, 46% were females and 54% were males. Average abundance of ticks per 100 m^2^ differed noticeably between regions (Fig. 2). Western Germany, Belgium and southern Sweden were regions with the highest and southern France and Estonia with the lowest average larval abundance (Fig. 2; Additional file 2). In all regions except Belgium, western Germany and central Sweden larvae made up less than 50% of the total number of collected ticks. The proportion of nymphs varied between 31% (western Germany) and 87% (southern France) of all ticks collected per region. Adults usually constituted less than 10% per region, except in Estonia and northern France, where they made up 54 and 21%, respectively.

During the tick surveys, the average air temperature ranged from 11.6 to 32.7 °C (median = 19.3 °C); soil temperature at 5 cm depth ranged from 7.6 to 18.7 °C (median = 13.8 °C); relative humidity at 5 cm height ranged from 31.7 to 99.9% (median = 74.2%) and from 25.8 to 99.7% (median = 63.8%) at 130 cm height.

### Model results

**Fig. 3:**
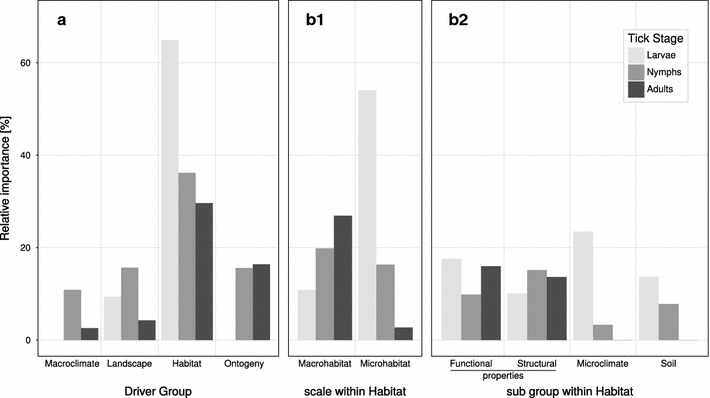
**a** Relative importance of groups of drivers in percent. Within the Habitat group, drivers were further grouped according to (**b1**) scale within habitat and (**b2**) sub groups. Relative importance is the relative contribution of all partial eta-squared (η^2^) values of a group to the total variation in the tick abundance data related to the fixed-effects part of the models

Across all ontogenetic stages, combined drivers of Macroclimate, Landscape, Habitat and Ontogeny had, on average, a relative importance of 4.2, 10.0, 44.7 and 10.1% respectively. However, the ontogenetic stages showed pronounced differences in all driver groups (Fig. 3). The overall model can be found in Additional file 9.

#### Larvae

Macroclimate did not have an effect on the abundance of larvae (Fig. 3).

**Fig. 4:**
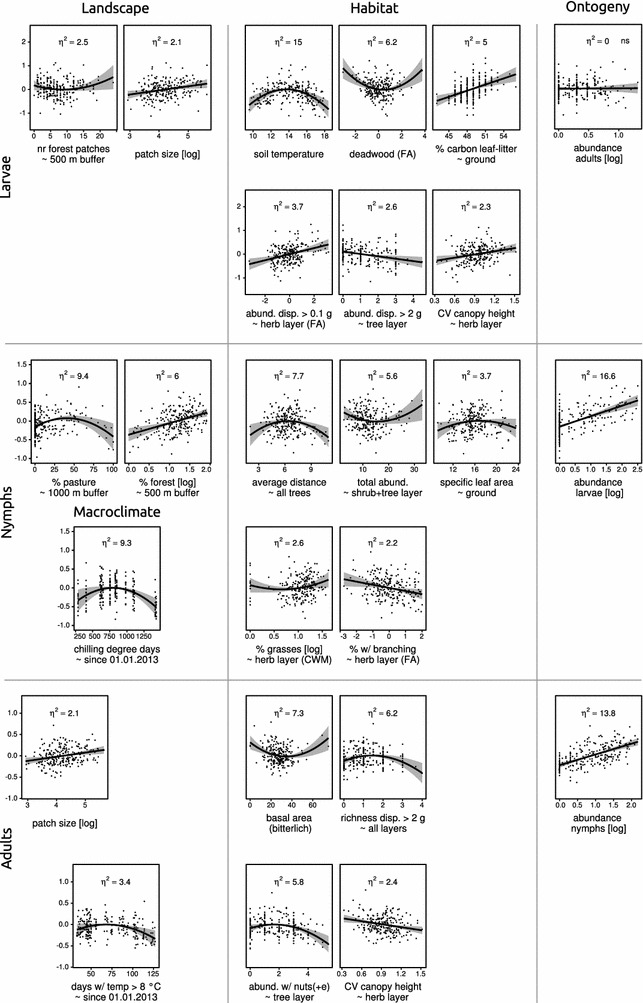
Subset of important response profiles. Each graph has a prediction line, confidence band (alpha = 0.05) and shows the partial residuals. The relative importance of the respective driver is given by partial eta-squared (η^2^). See Additional file 9 for full response profiles, *ns* not significant, *disp*. dispersules, *…(+e)* also including evergreen species, *abund*. abundance, *temp* temperature, *FA* correlation factor, *CV* coefficient of variation, *CWM* community weighted mean

The combined contribution of Landscape variables on larval abundance was only moderate (9.2% of the total variation) (Fig. 3). The number of forest patches within a 500 m buffer around focal patches (2.5%) and patches size (2.1%) were the most important drivers of this group (Fig. 4), the latter being positively correlated with larval abundance. Moreover, we found a small negative relationship of larval abundance with the proportion of pastures at landscape scale and a small positive relationship with agricultural cover at local scales (Additional file 10B).

Drivers of Habitat had a large effect on larval abundance, explaining together 64.8% of the total variation. Drivers associated to the Microhabitat made up 53.6% of the variation whereas Macrohabitat accounted for 10.7%. Microclimatic variables had the greatest contribution (22.6%), with soil temperature explaining 15%, and relative air humidity explaining 8% of the total variation. In both cases we found a unimodal, hump-shaped relationship (Fig. 4; Additional file 10D). Functional properties of the habitat explained 17.8% with several drivers related to the abundance of dispersules showing a positive effect on larval abundance. Only heavy dispersules showed a negative relationship (Additional file 10C). The soil (13.6%) and structural properties of the forest stand (10.0%) followed in importance. For example, there was a positive effect of the proportion of carbon in leaf-litter (Fig. 4), as well as of tree species richness on larval abundance (Additional file 10C). Structural properties as drivers were mainly associated with the understory layers (microhabitat). For instance, deadwood (6.2%) and the coefficient of variation of the potential height of plants in the herb layer (2.3%) partially contributed to larval abundance.

The abundance of adult ticks (Ontogeny) found in the patches, which was considered to be the previous ontogenetic stage for larvae, had no significant effect (Fig. 4).

#### Nymphs

The combined effect of Macroclimate had a low influence on the abundance of nymphs, with a relative contribution of 9.3% of the total variation (Fig. 1). The number of chilling degree days had a unimodal, hump-shaped effect on the abundance of nymphs (Fig. 4).

The driver group Landscape accounted for 15.5% of the total variation in nymphal abundance. The proportion of pasture within a 1000 m buffer had the largest effect within this group (9.4%) making it, together with the chilling degree days, the second most important single driver of the overall model. More nymphs were generally found in landscapes with a higher percentage of forest cover (Fig. 4).

Drivers of Habitat determined nymphal abundance to a relatively large degree (36.6%, Fig. 3). For nymphs, drivers of the Microhabitat added up to 15.6% and Macrohabitat accounted for 20.6%. Structural properties explained 15.6% of the total variation and this was mostly due to the average distance between trees (7.7%) and the total abundance of trees and shrubs (5.6%, Fig. 4). Functional properties were more important (9.9%) than soil conditions (8.4%) and functional properties were mostly found in the microhabitat of ticks. For instance, if berries were present in the tree-layer, their abundance correlated positively with nymphs (Additional file 11C). Microclimate explained a minor fraction (2.8%) of the total variation in nymphal abundance.

The abundance of larvae (Ontogeny) was the most decisive driver (16.6%) of nymphal abundance, with a strong positive effect (Fig. 4).

#### Adults

Variables of Macroclimate together explained only 3.4% of the total variation in adult abundance (Fig. 3), with number of days above 8 °C since the 1st of January being the only significant driver, with a hump-shaped relationship (Fig. 4).

Overall, the combined contribution of Landscape variables was minor (5.3%). The patch size was the most important driver (2.1%) and had a positive effect. Similar to the results for nymphs, an increase in overstory cover was also associated with higher adult abundance, although this effect was not strong (Additional file 12C). Across all patches, fewer ticks were found in landscapes with higher percentage of agricultural land-use.

Habitat was the most important group of drivers for adult ticks (combined 32.6% of the total variation, Fig. 3). Drivers of the Microhabitat made up 2.4% and Macrohabitat accounted for 27.2% of the total variation in the abundance of adults. Functional properties (16.4%) such as the richness of dispersules heavier than 2 g (6.2%) and the abundance of plants with nuts in the tree layer (5.8%), both with a hump-shaped relationship, were shown to be important drivers of adult abundance. Stand structural properties, explaining 13.1%, were slightly less important than functional properties. Basal area (7.3%) was the second most important single driver, although this relationship was largely driven by a few, extreme values (Fig. 4). The abundance of species producing nuts in the shrub layer, potentially providing food for tick-hosts, was positively correlated with adult tick abundance. Microclimatic variables and soil conditions played no role for adult abundance.

The abundance of nymphs (Ontogeny) was the most important single driver (13.8%) determining the abundance of adults, again with a positive relationship (Fig. 4).

## Discussion

We showed that numerous single drivers only had a moderate or small (<5% of the total variation) effect on abundance within each ontogenetic stage. This reflects the complex ecology of *I. ricinus*. Only some drivers, which exceed 5% of the total variation, are of major importance. However, combining single drivers into related groups, our results demonstrate that foremost drivers of the Habitat group and hereafter the Landscape group explained most of the total variation in abundance across all stages. Macroclimate on local scale explained only a minor part of the total variation for nymphs and adults, and no variation of the abundance of larvae.

### Macroclimate vs. habitat

Seasonal population dynamics (i.e. phenology) of all ontogenetic stages are largely dependent on temperature sums driving the tick’s developmental processes [17]. Regions with different annual climatic patterns lead to different temperature sums following the course of the year and this leads to region-specific macroclimatic effects on the potential tick abundance [13, 17, 56]. Window nested into region was used as random term in our LMMs and accounts for regional differences in tick response to all environmental conditions we did not record and which may correlate with region [16]. The optimum type (i.e. hump shaped) response of nymphs and adults to temperature accumulation indicates a macroclimatic niche for nymphs and adults. Obviously, low and high temperatures are detrimental to ticks, presumably due to either slow development or desiccation, respectively [17]. As we tested various macroclimatic metrics and found no significant effects otherwise, it shows that temperature sums are likely the most relevant macroclimatic drivers of tick abundance at local scales, when simultaneously considering other drivers such as Landscape, Habitat and Ontogeny.

Larvae dwell close to the forest floor [34], where macroclimate is buffered by the habitat properties [21]. This is consistent with our result showing that the combined effect of microclimate and other microhabitat properties explain the largest part of the total variation in larval abundance (Fig. 3). Even though macroclimate had an effect on the abundance of nymphs and adults, the effects of the abundance of shrubs and trees were also significant, suggesting a buffering by the habitat, both with respect to daily fluctuations and average values [21]. In answer to the first research question we thus conclude that Habitat properties, which buffer the macroclimate, have a considerable influence on tick abundance.

The suspected confoundedness between habitat and macroclimate is critical for the interpretation of the different driver groups, because macroclimatic factors modify and determine the habitat. For example, thermophilization, where warmer temperatures lead to an increasing dominance of warm-adapted plant species, may modify the tick microhabitat [57]. This is driven by climate buffering due to macrohabitat properties, such as the canopy cover [57, 58]. Microhabitat, as a place for tick questing, is hence indirectly modified, mediated by the abundance/cover of vegetation or the habitat type. It moreover becomes clear that ‘habitat suitability models’, which are often solely based on macroclimate properties, should be complemented with actual habitat related variables [16, 59]. For that matter, the largest part of climate stations are, by definition, located in open, non-forested sites [60], which rarely support high tick abundance [35], and are therefore not suitable to model the bioclimatic envelopes of forest-dwelling species like ticks [61].

### Habitat fragmentation

No clear effects of forest fragmentation or connectivity (higher edge density or nearest neighbor distance and lower proximity index) on tick abundance were observed. This is in contrast to what has been reported earlier [28, 30]. Forest fragmentation per se and habitat loss are two distinct processes [62], which often correlate but differ in their functional meaning [24]. Additionally, forest fragmentation creates habitat (edges and ecotones between habitat types), which is suitable for a variety of tick-host species [23], such as roe deer (*Capreolus capreolus*) and various small mammal species [30, 63]. This process of habitat creation for tick-hosts presumably has positive cascading effects on tick abundance.

The landscape metrics indicating forest fragmentation correlate with the proportion of landscape covered by forest (Additional file 4). The effects of habitat loss on the abundance of ticks can hence not clearly be disentangled statistically from habitat fragmentation per se. This finding, that more forest cover in the surrounding landscape increase tick abundance of all stages, seems to support the ‘amount of suitable habitat’-hypothesis [23] for ticks and tick-hosts (Fig. 4). The first part of our second research question can, however, neither be clearly negated, nor supported.

We also found an adverse effect on tick abundance, brought about by an increase in non-forest land-use types such as pastures or arable lands, which are considered unsuitable to most tick-hosts, corroborating once more the assumption of habitat amount as driving factor. In answer to the second part of our second research question, we found that landscape composition in terms of land-use classes other than forest, surrounding habitat patches, is a stronger driver of tick abundance than forest fragmentation per se. These effects may be based on influencing movement of tick-hosts [64], because pastures and arable land are unsuitable as habitat for various tick-hosts.

### Relevant habitat properties

As a response to our third research question, we found that structural and functional properties of forest stands were more important than the potential effects of historical continuity of the stand. In fact, patch age was not significant at all. In contrast to our expectation, habitat diversity had only a minor relative importance on the abundance of all ontogenetic tick stages (second part of our third research question). Apparently, the presence of certain structural (and morphological) or functional properties is more important for the tick cycle, than the diversity of such elements.

Many of the macrohabitat related drivers are indirect, mediated by tick-hosts (in fact this indirect nature is a premise for a habitat driver to also be considered a macrohabitat driver). For example, there is no known direct link between ticks and dispersules other than tick-hosts feeding on dispersules and hosting ticks at the same time [22, 65]. Dispersules, as food resource for tick-hosts, do play a considerable role in explaining tick abundance. The fact that we found an effect of not only nuts, but also berries as important dispersules, suggests that birds and not only small mammals are tick-hosts in fragmented forest patches. The relative importance of functional habitat properties is on par with that of structural properties. Many stand structural properties are indirect drivers because they indicate the provision of shelter for tick-hosts or, as outlined above, are related to buffering the macroclimate and hence the creation of more suitable ambient conditions for ticks.

Many of the microhabitat drivers are directly related to the survival and questing success of ticks. An effect of plant communities and associated soil and microclimate conditions on tick abundances has for example been shown by [11] and [66]. Several of our potentially driving variables were comprised in the categorical variable *plant community* in these studies. In our models we showed that several of these plant community associated drivers, such as herb-layer properties, microclimate and soil conditions generally drive tick abundances at all ontogenetic stages, also when retained simultaneously in a model as distinct variables (in contrast to as plant community in [11]). While ontogenetically older ticks often quest in higher vegetation layers [34], they can relocate, if their preferred questing habitat shows less suitable climatic conditions [36]. Since larvae dwell in or close to the forest floor without being able to climb into higher vegetation due to faster desiccation there, they can often not evade climatically adverse conditions [36]. It is hence not surprising that leaf litter, soil conditions and particularly microclimate are amongst the most important drivers o larval abundance compared with nymphs and adults (Fig. 3).

### Niche differences between ontogenetic stages

With respect to our fourth research question, we found clear differences in drivers (Fig. 4; Additional file 13) between the different ontogenetic stages of *I. ricinus* and also a positive effect of the previous stage’s abundance for nymphs and adults. The result that larvae were not driven by adults is somewhat trivial and may depend on their aggregated occurrence. It is known that ontogenetic stages of ticks occupy different niches [34, 36]. The relatively large effect of the preceding stage indicates that a certain part of the total variation in one stage’s abundance can be explained indirectly by the drivers of the previous stage’s abundance. This indicates general habitat suitability for ticks or their hosts and hence that ticks are affected by similar drivers to the degree of the effect size of the previous stage’s abundance. It is however not clear if this is due to effects of harmonized local survival of the respective stages or due to habitat suitability for tick-hosts, which constantly disperse new ticks to this habitat patch. Both, survival and tick-host suitability may interact with ontogeny, because with increasing ontogenetic age ticks are driven increasingly by the macrohabitat (Fig. 3a), which is associated to the carrying capacity of the habitat patch towards tick-hosts [67, 68].

Such ontogenetic niche differentiation is scale-dependent. At the habitat scale, the developmental stages occupy different height strata for questing. This vertical niche differentiation is a result of the stage-specific requirements for microclimatic conditions and physical structures for questing. An ontogenetic niche differentiation additionally extends at the landscape scale, because of the host-mediated movement of ticks (Fig. 1), which depends on the life-history traits of the host. As the ontogenetic stages are restricted to different hosts to a certain degree, they may be transported from several hundreds of meters (e.g. larvae and nymphs on resident hosts such as small mammals within a habitat patch) up to tens of kilometers (nymphs and adults on dispersal hosts such as birds or ungulates within a landscape) [69]. However, knowing the resident-to-dispersal hosts ratio in response to habitat properties poses one recent challenge. We should nevertheless consider a pronounced ontogenetic niche differentiation, which may not only be relevant for different heights of questing within one habitat, but could as well extend to landscape scale. This may have important implications for the dynamics of *Borrelia burgdorferi* genospecies, which are associated to specific host groups [70, 71].

## Limitations of our study

We are aware that our sampling of ticks is biased as it does not consider a region-specific life cycle, which might have an unimodal but also bimodal distribution of tick abundance throughout the year in different regions [13, 17, 56]. Such a snapshot does not consider inter-annual changes in the role of the environmental drivers, which may well be important. By collecting ticks only once in each patch, smaller patches may not be as well represented as larger patches, because a higher probability of uncontrolled-for variation sources can influence the measurement here. However, by collecting ticks in a window within 1 week, we ensured that tick numbers were comparable within each region, irrespective of their current mode of activity or macroclimatic events. This allowed us to test for the relevance of drivers within each region. In addition, the random effect in our LMMs statistically accounts for variation accountable to regions and makes the other environmental predictors comparable across regions. Nevertheless, studying population dynamics, with a higher sampling effort over time in each region will certainly reveal additional, region specific trends. The trends we revealed are valid for the overall studied (European) gradient representative of lowland agricultural landscapes.

Finally, various herb layer characteristics may influence the sampling success of the cloth dragging method, possibly obscuring the true abundance of ticks, particularly larvae [39]. For instance, dense vegetation may limit the detection of larvae and nymphs questing close to the ground. We did, however, not find a negative effect of general vegetation density, but of the proportion of herbaceous plants with ascending or prostrating habitus. These plants form a denser layer of vegetation, sometimes even with thorns, which is basically impenetrable with the cloth. Although we deliberately avoided such sites, even small differences in the abundance of such plant species could potentially affect the sampling effectiveness. Nevertheless, there is no reason to assume that the sampling success of the cloth dragging method was systematically correlated with most of the selected driver variables, suggesting that our results are rather robust with respect to the sampling procedure.

## Conclusions

Abundance of *I. ricinus* in forest fragments of agricultural landscapes is shaped by a rather large number of drivers and this emphasizes the complex ecology of ticks. Even though most drivers had a modest to small explanatory power, their combined effect was substantial. We showed that landscape and particularly habitat quality play an important role, outweighing the effects of macroclimate by far to explain local variation in tick abundance.

These patterns may, however, be context specific. The studied forest patches comprise one relatively similar type of tick habitat. A wider range in various environmental gradients and in habitat types beyond forest, where ticks are also present (grasslands, hedgerows, wetlands, etc.) is required to reliably quantify the niche of *I. ricinus*.

Further investigations on the influence of forest and landscape management on ticks are urgently required to disentangle the influence of anthropocentric management decisions impacting the abundance of *I. ricinus,* and hence the ecosystem disservice of TBD prevalence. Such management decisions affect a large variety of the tick drivers we identified here, including the amount of suitable forest and ecotone habitat, the cover and composition of the herb layer, and hence the habitat and resource availability for tick-hosts.

Certainly, habitat and landscape management effects should be separated from effects of climate change, which are largely out of reach of direct human ecosystem management. However, climate only has a relatively small explanatory value on local scales, when accounting for the effect of habitat properties. The local scale is what really matters for the contact between ticks and humans and effects of climate change may thus be less important than ecosystem management for TBD risks in humans. We suggest that drivers that can be, and were historically, manipulated by humans have a lot larger relative importance than assumed before.

## Additional files

**Additional file 1.** Meta-data on study setup. A table outlining the most important meta-data, such as center of each studied landscape window, dates of sampling, number of forest patches and a couple of landscape metrics to characterize the respective landscapes.

**Additional file 2.** Focal variables as drivers of tick abundances. An extensive list of all focal variables considered in this study. They are classified according to the driver groups outline in the methods section.

**Additional file 3.** Methods – technical details. The text outlines some technical details of the methods. These descriptions help understanding in detail what has been done, but are not necessary to understand what has been measured.

**Additional file 4.** Correlation factors. The text-file describes how correlation factors were derived and what the name components of our internal variable names mean. The spreadsheet presents the loadings of all correlation factors, which are above |0.5| and hence indicate that the respective variable is correlated to the correlation factor.

**Additional file 5.** Refer to the caption of Additional file 4.

**Additional file 6.** Outline of model-building procedure (also Additional file 7). Text outlining in detail the model building/variable selection procedure. The figure presents a flow chart of the different steps taken.

**Additional file 7.** Flow chart of data preparation and model selection procedure.

**Additional file 8.** Equations to calculate effect sizes for significant drivers of tick abundance.

**Additional file 9.** Overall response profiles and model-output (also Additional files 10, 11, 12). Figure for each tick stage, outlining the response for its respective linear mixed model. Each graph has a prediction line, confidence band (alpha = 0.05) and shows the partial residuals. η^2^ represents the relative importance of the respective driver.

**Additional file 10.** Response profiles for larval abundance. Each graph has a prediction line, confidence band (alpha = 0.05) and shows the partial residuals. Drivers of (A) Macroclimate (no significant effects) (B) Landscape, (C) Macrohabitat, (D) Microhabitat, (E) Method control, (F) Ontogeny. ‘ns’ = not significant, ‘abund.’ = abundance, ‘disp.’ = dispersules, ‘asc./pro. hab.’ = ascending or prostrating habitus, ‘reg. leaf-dist.’ = leaf distribution regular on stem, FA = correlation factor, CV = coefficient of variation, CWM = community weighted mean.

**Additional file 11.** Response profiles for nymphal abundance. Each graph has a prediction line, confidence band (alpha = 0.05) and shows the partial residuals. Drivers of (A) Macroclimate (B) Landscape, (C) Macrohabitat, (D) Microhabitat, (E) Method control, (F) Ontogeny. ‘ns’ = not significant, ‘abund.’ = abundance, ‘tree1/tree2’ = upper/lower tree-layer, FA = correlation factor, CWM = community weighted mean.

**Additional file 12.** Response profiles for adult abundance. Each graph has a prediction line, confidence band (alpha = 0.05) and shows the partial residuals. Drivers of (A) Macroclimate (B) Landscape, (C) Macrohabitat, (D) Microhabitat, (E) Method control, (F) Ontogeny. ‘ns’ = not significant, ‘disp.’ = dispersules, ‘…(+e)’ = also including evergreen species, ‘abund.’ = abundance, ‘temp’ = temperature, CV= coefficient of variation.

**Additional file 13.** Overall relative importance incl. sampling method. Similar to Fig. 3, this graph shows the relative importance values of all significant drivers, when variables controlling for the method are included in the model. Relative importance of categories of drivers in percent, including the relative importance of metrics capturing the impact of our method. See Fig. 3.
