# Supplementary material for: Environmental drivers of Ixodes ricinus abundance in forest fragments of rural European landscapes
Source: BMC Ecol. 2017 Sep 6;17:31. doi: 10.1186/s12898-017-0141-0 (PMC5586062; doi:10.1186/s12898-017-0141-0)
Supplement: Supplementary file 3 — Additional file 3. Methods – technical details. The text outlines some technical details of the methods. These descriptions help understanding in detail what has been done, but are not necessary to understand what has been measured. [file 12898_2017_141_MOESM3_ESM.docx]

**Tick survey**

Rain started on a few occasions only (1% of drags) and sampling was finished under rainy conditions in these plots and continued in other plots after rain stopped. After sampling, the wetness of the cloth was estimated with the categories 'dry' (74.0% of drags), 'bottom moist' (10.4%), 'moist' (13.0%), 'bottom wet' (0.5%) and 'wet' (2.1%). The category 'moist' was assigned when the first signs of water (e.g. drops from the vegetation) were perceptible on the flag. As all values were collected in a plot and subsequently averaged per patch, also the wetness of the cloth was treated like that. We assigned numerical values to each of the categories and averaged these over patches. After this, 77% of patches (190) had an average value = 1, which was assigned to a dry flag. Another 20.6% of patches (51) had a value > 1 and <= 3, which were assigned to drags which were at the bottom or overall moist. The remaining 2.4% of patches (6) were sampled with a wet or partially wet cloth. Of these, 5 patches were in southern France. These averages reflect quite well the plot averages, even though such a procedure is strictly speaking not allowed for categorical values. Non-integer average values indicate that a patch was sampled with a different cloth wetness in its plots, but these cases are relatively rare (7.5%). We tested the final model while including the categories for wetness and also the time of the day as fixed effects to correct for these effects, in case they had an influence. The adult stage was the only stage where the abundance decreased as flag wetness increased (1.0% relative importance) and with time of sampling later in the day (2.1%). Consequently we only present the models excluding these methodological control variables.

Sampling of ticks occurred within maximum one week with only one exception. In Belgium heavy rains set in shortly after sampling started and we hence moved to another region and continued sampling of the Belgian landscape windows about a month later.

**GIS work**

Landscapes were characterized utilizing ArcGIS 10.3 (ESRI 2014), based on maps acquired within the framework of smallFOREST [1]. Maps were digitized from recent aerial photographs and historical maps of the last centuries. Buffers for land-use types and hedgerows were calculated as concentric circles between 0 – 50 m, 50 – 100 m, 100 – 250 m, 250 – 500 m, 500 – 1000 m and 1000 – 5000 m. Edge density and edge habitat area was calculated only in the 100 – 250 m and 250 – 500 m buffers. Proximity index was calculated for a maximum distance of 5000 m.

**Patch Age**

The patch age is calculated as average of the century of the map, where a sub patch emerged weighted by its proportion of the recent patch [1].

**Forest stand characterisation**

The Point-Center-Quarter (PCQ) method [2] was used to select trees for measuring stand structure. Two trees per quarter were measured, so that they would be of small and large diameter to represent different layers or age classes. Small trees were chosen to be between 7 cm and 30 cm in diameter at breast height (D_130_) and large trees were larger than 30 cm at D_130_. In addition to the PCQ survey, four neighbouring trees were measured (“structural group of four”, [3]). The tree closest to the plot centre was chosen as reference tree and the same set of variables, except tree height were determined.

**Soil Analysis**

Bulkdensity was estimated from the soil samples and used to quantify the pools of C and N after chemical analysis. The samples were dried at 40 °C and weighed. The soil was passed through a 1 mm sieve to remove stones and gravel. Soil pH (CaCl_2_) was measured using a glass electrode (Orion, Orion Europe, Cambridge, England, model 920A) following the procedure described in ISO 10390:1994(E). The concentration of C and N was measured by high temperature combustion at 1150°C using an elemental analyser (Vario MACRO cube CNS, Elementar, Germany). After complete destruction with HClO_4_ (65%), HNO_3_ (70%) and H_2_SO_4_ (98%) in Teflon bombs for four hours at 150 °C total P concentrations in mineral soil were measured according to the malachite green procedure [4].

**Microclimate**

For analysis, air temperature at 5 cm and 130 cm height were averaged, since they correlated highly (r = 0.92) and did not differ significantly (t-test, p = 0.5). Relative humidity values were also correlated (r = 0.75), but did differ between 5 cm and 130 cm height (t-test, p < 0.001) and were thus treated separately.

**Bibliography**

1. Valdés A, Lenoir J, Gallet-Moron E, Andrieu E, Brunet J, Chabrerie O, et al. The contribution of patch-scale conditions is greater than that of macroclimate in explaining local plant diversity in fragmented forests across Europe: Drivers of herbaceous species diversity in fragmented forests. Global Ecology and Biogeography. 2015;24:1094–105.

2. Cottam G, Curtis JT. The Use of Distance Measures in Phytosociological Sampling. Ecology. 1956;37:451–460.

3. Pommerening A. Approaches to quantifying forest structures. Forestry. 2002;75:305–324.

4. Lajtha K, Driscoll CT, Jarrell WM, Elliott ET. Soil phosphorus. Characterization and total element analysis. In: Robertson GP, Coleman DC, Bledsoe CS, Sollins P, editors. Standard soil methods for long-term ecological research. Oxford University Press; 1999. p. 115–42.
