# Supplementary material for: Environmental drivers of Ixodes ricinus abundance in forest fragments of rural European landscapes
Source: BMC Ecol. 2017 Sep 6;17:31. doi: 10.1186/s12898-017-0141-0 (PMC5586062; doi:10.1186/s12898-017-0141-0)
Supplement: Supplementary file 6 — Additional file 6. Outline of model-building procedure (also Additional file 7). Text outlining in detail the model building/variable selection procedure. The figure presents a flow chart of the different steps taken. [file 12898_2017_141_MOESM6_ESM.docx]

The overall statistical procedure is captured in Fig. A5. We started with an intercept-only model. For all variables we calculated the model performance when adding the respective variable to a successively growing model. Model performance was represented by multiple measures, namely AIC, the significance level of the variable in the resulting model and the coefficient of determination of a simple linear model with the variable as response to the so far selected model without this variable (R²_V_). R²_V_ quantifies how strong the variable would be explained by the preexisting model and thus how strong covariance between the preexisting model and the respective variable would be. We filtered variables with R²_V_ <= 0.5 to have only variables without covariance remaining. More significant variables were preferred over less significant variables. When firstly significance levels and then R²_V_ of several variables were similar, we chose the ecologically more meaningful and easier to interpret variable. We checked partial residual plots, marked data-points with an out-layer factor of more than 3 standard deviations from the mean thereof, plotted linear and polynomial trend lines for both, all data points and the subset of non-outliers and assured like that visually that a variable was not chosen based on influential outliers only. The value of AIC was constantly observed and it was assured that the overall model does not “drift away” (= the AIC became worse and worse with each iteration). We repeated this process until no variables fulfilling the above described characteristics remained. Questionable or surprising (i.e. ecologically hard to interpret) variables, in response to the given model, were kept out of the model and only included, if they repeatedly reemerged as “suitable variable”.

[Fig. A5 here]
