# Supplementary figures and images for: Environmental drivers of Ixodes ricinus abundance in forest fragments of rural European landscapes
Source: BMC Ecol. 2017 Sep 6;17:31. doi: 10.1186/s12898-017-0141-0 (PMC5586062; doi:10.1186/s12898-017-0141-0)

## Data Preparation

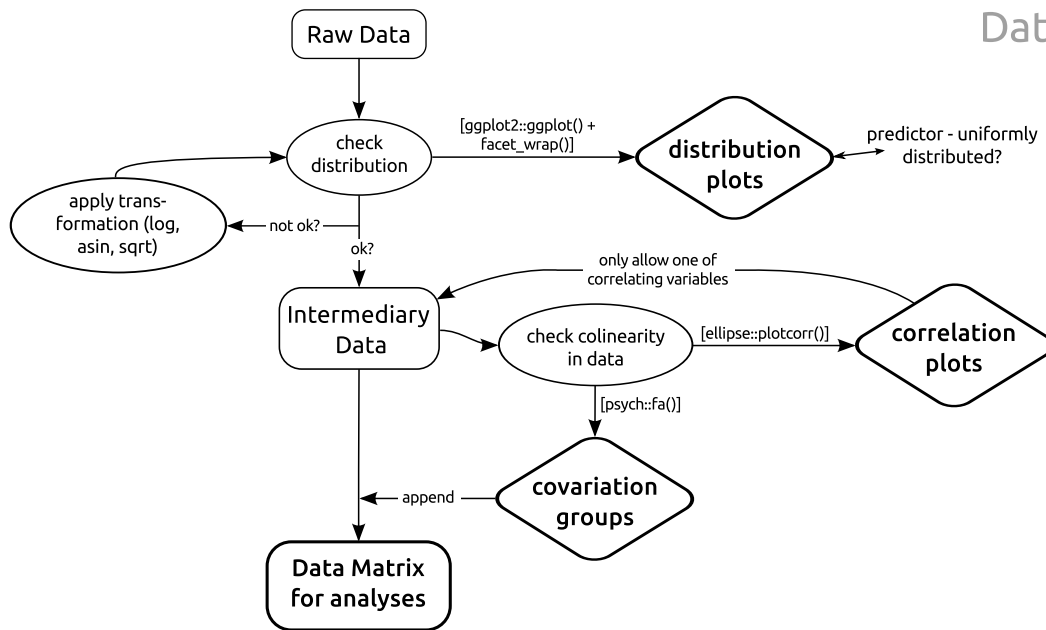

## Model Building

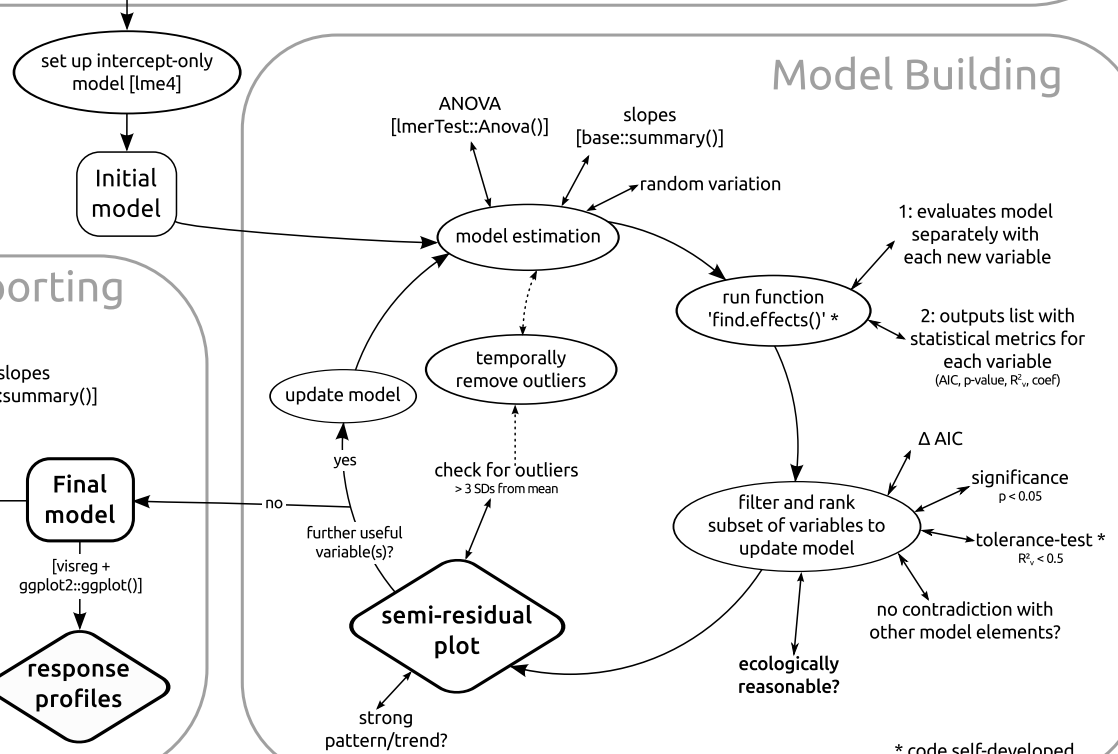

## Reporting

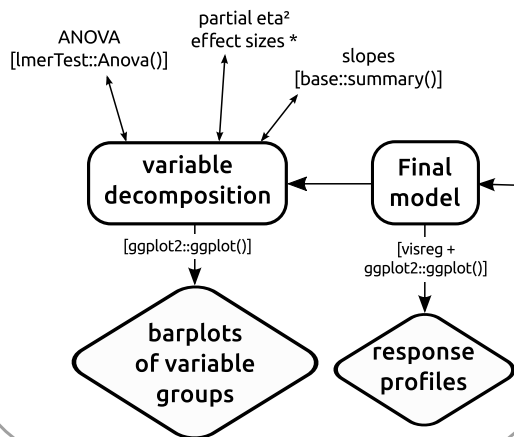

\* code self-developed

Supplement: Supplementary file 7 — Additional file 7. Flow chart of data preparation and model selection procedure. [file 12898_2017_141_MOESM7_ESM.pdf]

# A no significant effects

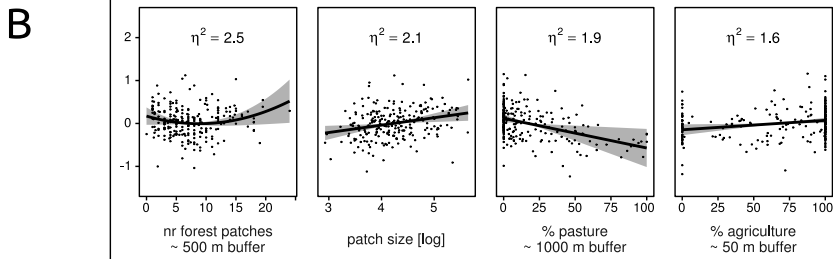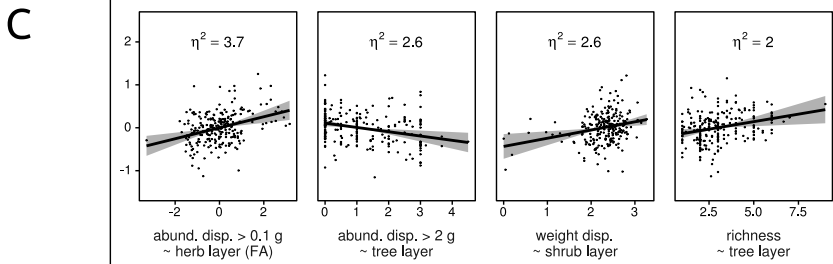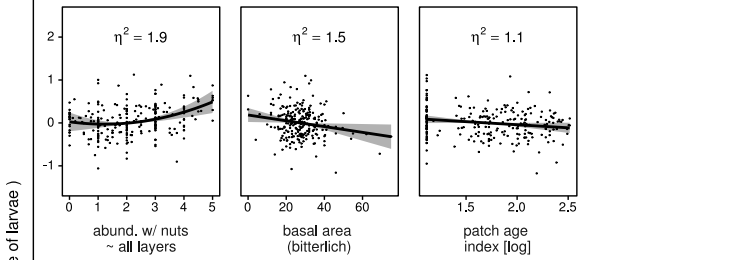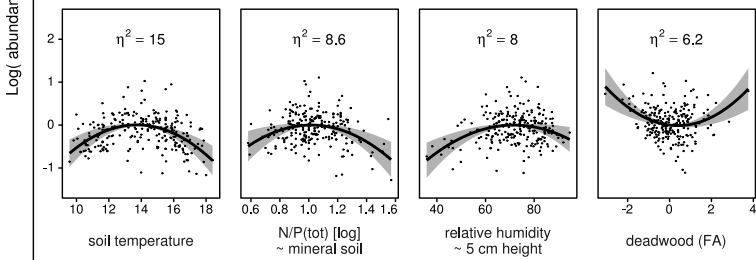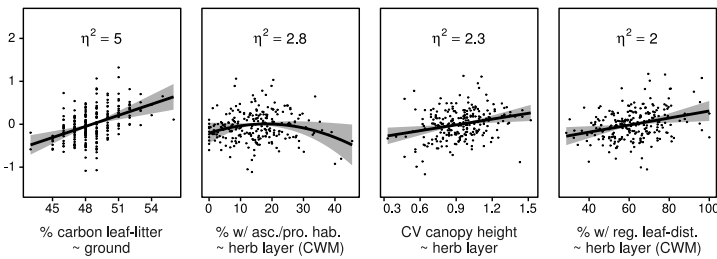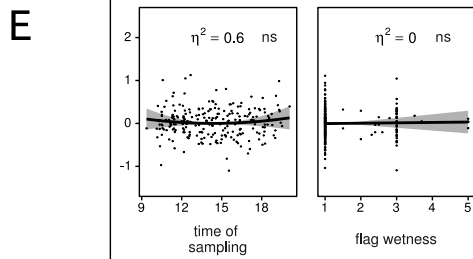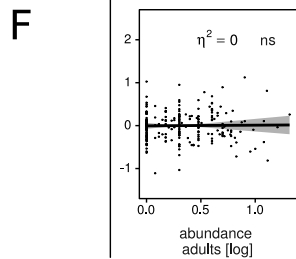

Supplement: Supplementary file 10 — Additional file 10. Response profiles for larval abundance. Each graph has a prediction line, confidence band (alpha = 0.05) and shows the partial residuals. Drivers of (A) Macroclimate (no significant effects) (B) Landscape, (C) Macrohabitat, (D) Microhabitat, (E) Method control, (F) Ontogeny. ‘ns’ = not significant, ‘abund.’ = abundance, ‘disp.’ = dispersules, ‘asc./pro. hab.’ = ascending or prostrating habitus, ‘reg. leaf-dist.’ = leaf distribution regular on stem, FA = correlation factor, CV = coefficient of variation, CWM = community weighted mean. [file 12898_2017_141_MOESM10_ESM.pdf]

A

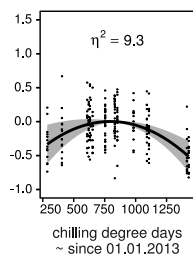

B

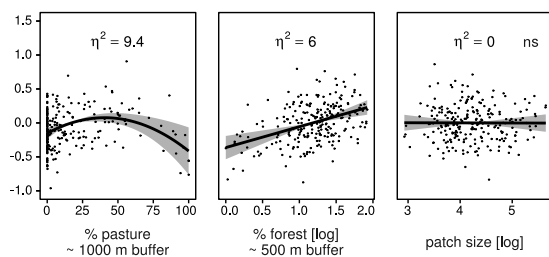

C

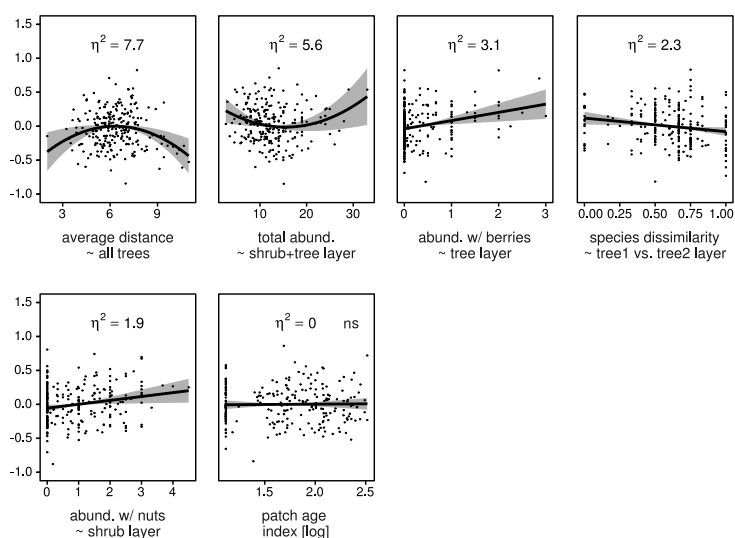

D

Log( abundance of nymphs )

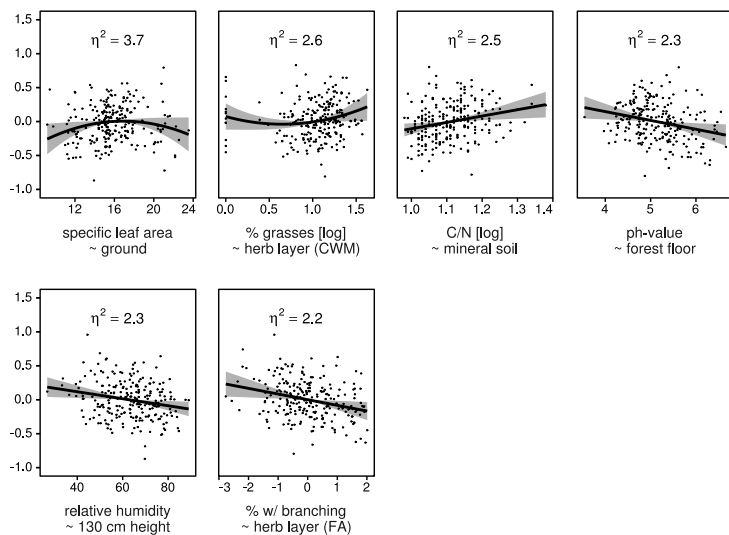

E

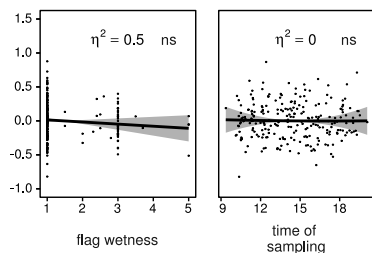

F

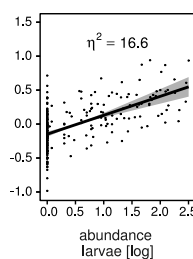

Supplement: Supplementary file 11 — Additional file 11. Response profiles for nymphal abundance. Each graph has a prediction line, confidence band (alpha = 0.05) and shows the partial residuals. Drivers of (A) Macroclimate (B) Landscape, (C) Macrohabitat, (D) Microhabitat, (E) Method control, (F) Ontogeny. ‘ns’ = not significant, ‘abund.’ = abundance, ‘tree1/tree2’ = upper/lower tree-layer, FA = correlation factor, CWM = community weighted mean. [file 12898_2017_141_MOESM11_ESM.pdf]

A

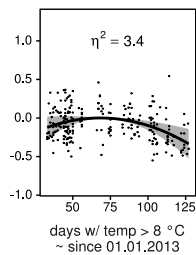

B

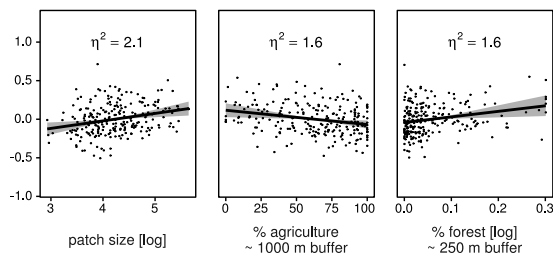

C

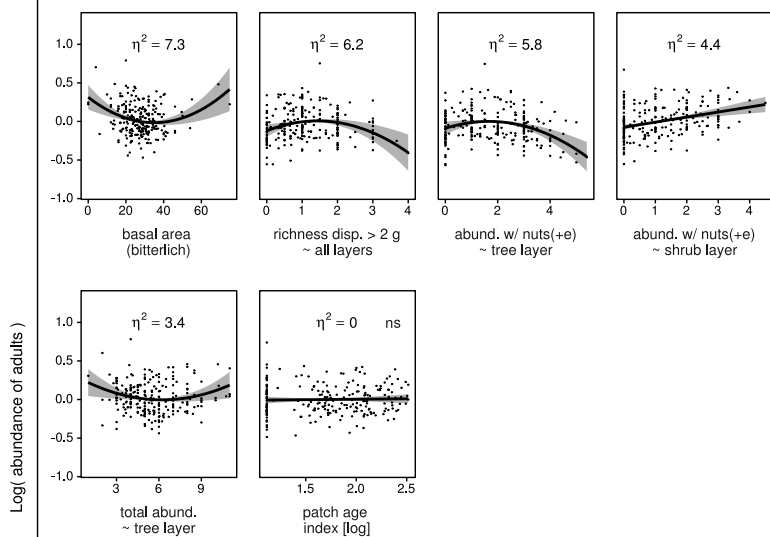

D

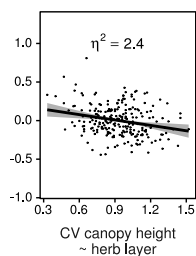

E

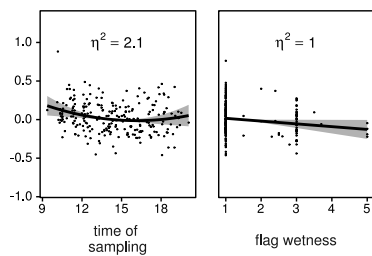

F

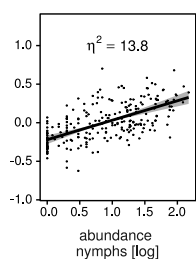

Supplement: Supplementary file 12 — Additional file 12. Response profiles for adult abundance. Each graph has a prediction line, confidence band (alpha = 0.05) and shows the partial residuals. Drivers of (A) Macroclimate (B) Landscape, (C) Macrohabitat, (D) Microhabitat, (E) Method control, (F) Ontogeny. ‘ns’ = not significant, ‘disp.’ = dispersules, ‘…(+e)’ = also including evergreen species, ‘abund.’ = abundance, ‘temp’ = temperature, CV= coefficient of variation. [file 12898_2017_141_MOESM12_ESM.pdf]

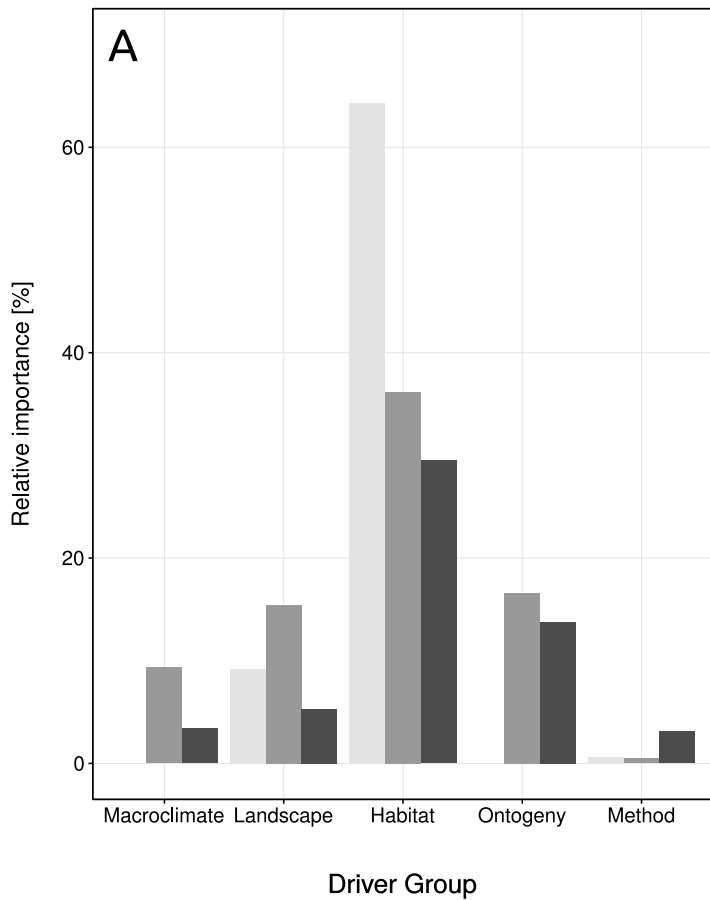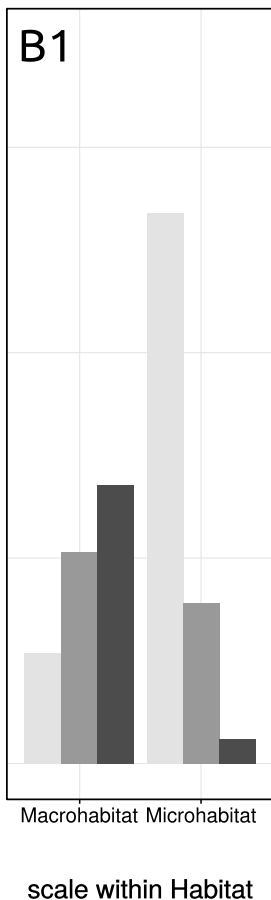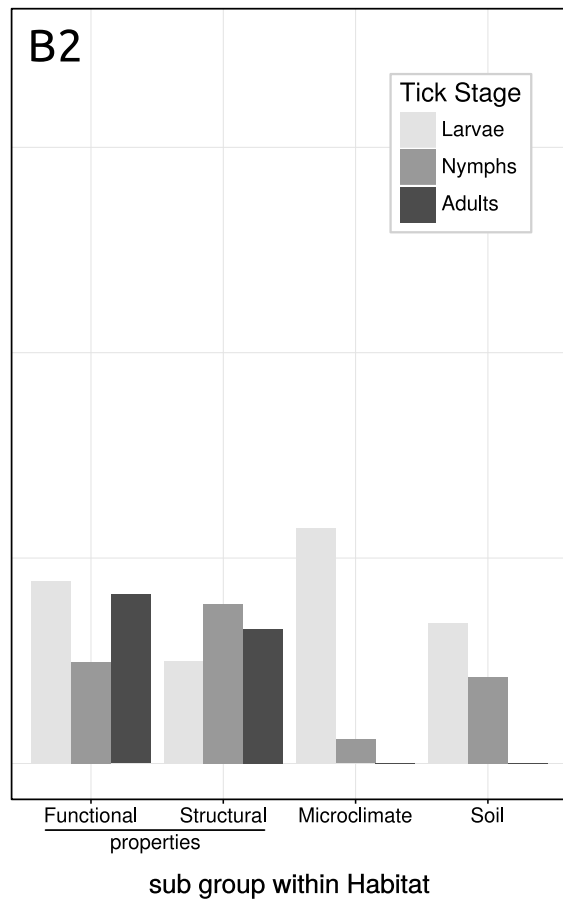

Supplement: Supplementary file 13 — Additional file 13. Overall relative importance incl. sampling method. Similar to Fig. 3, this graph shows the relative importance values of all significant drivers, when variables controlling for the method are included in the model. Relative importance of categories of drivers in percent, including the relative importance of metrics capturing the impact of our method. See Fig. 3. [file 12898_2017_141_MOESM13_ESM.pdf]
