# Supplementary material for: Environmental drivers of Ixodes ricinus abundance in forest fragments of rural European landscapes
Source: BMC Ecol. 2017 Sep 6;17:31. doi: 10.1186/s12898-017-0141-0 (PMC5586062; doi:10.1186/s12898-017-0141-0)
Supplement: Supplementary file 8 — Additional file 8. Equations to calculate effect sizes for significant drivers of tick abundance. [file 12898_2017_141_MOESM8_ESM.docx]

As effect size, we calculated partial eta²:

$\eta_{\delta i}^{2}=\frac{{SS}_{\text{fixed i}}}{{SS}_{\text{fixed i}}+{SS}_{error}}$ (1)

with SS_fixed i_ = i-th fixed effect sum of squares, SS_error_ = residual sum of squares (Richardson 2011). From this, we derived relative importance as fraction of each partial eta² of the sum of all partial eta² values and scaled to the R²_adj_ of fixed effects:

$R{^{2}}_{\text{adj fixed}}=1-\frac{\left( {SS}_{total}-\sum_{i=1}^{p} {SS}_{\text{fixed i}} \right)/\left( n-p-1 \right)}{{{SS}_{total}}/\left( n-1 \right)}$ (2)

with SS_total_ = total sum of squares, n = number of observations, p = number of explanatory variables.

${relImp}_{i}=\frac{\eta_{\delta i}^{2}}{\sum_{i=1}^{p} \eta_{\delta i}^{2}}\cdot R_{\text{adj fixed}}^{2}$ (3)

with η_δi_^2^ = partial eta² of the i-th explanatory variable, p = number of explanatory variables and R²_adj fixed_ = R^2^_adj_ of fixed effects.

This is necessary, because partial eta² values can not directly be used as measures of percentage of the total variance, in contrast to eta², which directly represents the proportion of the total variance (Richardson 2011). However, partial eta² is preferable over eta², because the effect of other independent variables and interactions are partialled out of the effect in focus (Richardson 2011).
